# Genomic measures of inbreeding coefficients and genome-wide scan for runs of homozygosity islands in Iranian river buffalo, *Bubalus bubalis*

**DOI:** 10.1186/s12863-020-0824-y

**Published:** 2020-02-10

**Authors:** Seyed Mohammad Ghoreishifar, Hossein Moradi-Shahrbabak, Mohammad Hossein Fallahi, Ali Jalil Sarghale, Mohammad Moradi-Shahrbabak, Rostam Abdollahi-Arpanahi, Majid Khansefid

**Affiliations:** 1 Department of Animal Science, University College of Agriculture and Natural Resources, University of Tehran, Karaj, 31587-11167 Iran; 2 Departments of Animal and Poultry Science, College of Aburaihan, University of Tehran, Pakdasht, 33916-53755 Iran; 3 AgriBio Centre for AgriBioscience, Agriculture Victoria, Bundoora, VIC 3083 Australia

**Keywords:** Water buffalo, River buffalo, Genetic diversity, Inbreeding, Gene enrichment, Runs of homozygosity, Selection signatures

## Abstract

**Background:**

Consecutive homozygous fragments of a genome inherited by offspring from a common ancestor are known as runs of homozygosity (ROH). ROH can be used to calculate genomic inbreeding and to identify genomic regions that are potentially under historical selection pressure. The dataset of our study consisted of 254 Azeri (AZ) and 115 Khuzestani (KHZ) river buffalo genotyped for ~ 65,000 SNPs for the following two purposes: 1) to estimate and compare inbreeding calculated using ROH (F_ROH_), excess of homozygosity (F_HOM_), correlation between uniting gametes (F_UNI_), and diagonal elements of the genomic relationship matrix (F_GRM_); 2) to identify frequently occurring ROH (i.e. ROH islands) for our selection signature and gene enrichment studies.

**Results:**

In this study, 9102 ROH were identified, with an average number of 21.2 ± 13.1 and 33.2 ± 15.9 segments per animal in AZ and KHZ breeds, respectively. On average in AZ, 4.35% (108.8 ± 120.3 Mb), and in KHZ, 5.96% (149.1 ± 107.7 Mb) of the genome was autozygous. The estimated inbreeding values based on F_HOM_, F_UNI_ and F_GRM_ were higher in AZ than they were in KHZ, which was in contrast to the F_ROH_ estimates. We identified 11 ROH islands (four in AZ and seven in KHZ). In the KHZ breed, the genes located in ROH islands were enriched for multiple Gene Ontology (GO) terms (*P* ≤ 0.05). The genes located in ROH islands were associated with diverse biological functions and traits such as body size and muscle development (BMP2), immune response (CYP27B1), milk production and components (MARS, ADRA1A, and KCTD16), coat colour and pigmentation (PMEL and MYO1A), reproductive traits (INHBC, INHBE, STAT6 and PCNA), and bone development (SUOX).

**Conclusion:**

The calculated F_ROH_ was in line with expected higher inbreeding in KHZ than in AZ because of the smaller effective population size of KHZ. Thus, we find that F_ROH_ can be used as a robust estimate of genomic inbreeding. Further, the majority of ROH peaks were overlapped with or in close proximity to the previously reported genomic regions with signatures of selection. This tells us that it is likely that the genes in the ROH islands have been subject to artificial or natural selection.

## Background

There are two main species of buffalo: the Asian water buffalo (*Bubalus bubalis*) and the African wild buffalo (*Syncerus caffer*), the second of which is also known as the cape buffalo [1, 2]. Domestication of *B. bubalis*, including of the river (*B. bubalis bubalis*, 2n = 50) and swamp (*B. bubalis carabanensis*, 2n = 48) subspecies, occurred approximately 3000–6000 years ago [3]. The domestication of river buffalo occurred in the Indo–Pakistani area, and domestication of swamp buffalo occurred close to the border of China [4]. River buffalo expanded broadly from India, Egypt and Southeast Asia to Europe, and the swamp buffalo is the most common type of buffalo in China and Southeast Asia [3–5]. The worldwide water buffalo population accounts for only approximately 11% of the entire cattle population. However, the population of water buffalo has increased in the past five decades by approximately 1.65% annually [5]. India, Pakistan and Europe (with 5.3, 4.8 and 4.5% rates of increase, respectively) have the highest rates of annual increase [6].

In many tropical and subtropical countries, river buffalo are raised for both meat and milk production [7]. In Iran and in other developing nations, river buffalo production is of great economic importance because of the ability of buffalo to make the best use of low-quality feed in the production of their valuable milk, which has a unique taste and curd properties, high resistance to local parasites, high adaptation to harsh climate conditions, and long productive lifespan. The three major Iranian river buffalo breeds are Azeri (AZ), Khuzestani (KHZ) and Mazandarani (MZ), and each of these breeds belongs to different geographical zone [2]. The AZ, KHZ and MZ are common in the north-west and north, west and south-west, and north of the country, respectively. In Iran, the recording of milk and meat production, and the selective breeding of buffalo for better dairy performance (i.e. in milk production, and fat and protein percentage) and better meat production are performed by the Animal Breeding Centre of Iran (ABCI) [2]. Following performance and pedigree recording in some herds, and genetic analysis, candidate bulls are selected from rural herds based on their genetic merits, and the semen of these selected bulls is collected and distributed to all herds [2]. However, despite buffalo production being important in Iran, particularly in rural regions, controlling inbreeding and ensuring genetic improvement of desired traits through traditional breeding programmes are difficult because of a shortage of reliable pedigree and performance records for water buffalo in the country.

The inbreeding coefficient measured from pedigree information (F_PED_) has been the most common parameter for describing the level of inbreeding since Wright [8] However, the reliability of the estimated F_PED_ depends on the completeness and correctness of pedigree. With the availability of high-density SNP-chip markers, inbreeding can also be defined according to genomic information such as genome-wide autozygosity [9] Autozygosity occurs when parents pass identical chromosomal fragments, which they already inherited from a common ancestor, on to their offspring [10]; these genomic regions of homozygosity are known as runs of homozygosity (ROH) [11, 12]. Estimated inbreeding based on ROH (F_ROH_) can discriminate between homozygous (i.e. identical by descent [IBD]) and non-autozygous (i.e. identical by state [IBS]) positions in the genome [9]. Further, well-recorded pedigree information is not required to have reliable F_ROH_. Thus, using genetic markers instead of pedigree information to calculate inbreeding can produce more robust estimates [13, 14].

Identifying ROH can also help to find the footprints of genetic selection on the genome [15–17]. However, ROH are suggestive, but not conclusive, of genomic regions under natural or artificial selection because the incidence, extent and distribution of ROH across the genome are influenced by many factors other than ROH, such as recombination rate, population structure, mutation rate and inbreeding [16]. Nevertheless, ROH that frequently occur among individuals may contain genes associated with different traits that have been under historical selection, so that the genes located in ROH islands can be important for selective breeding [13, 15, 18]. ROH can also provide detailed information on the genetic relatedness of animals, which allows breeders to better control inbreeding in the population [16]. This allows mate allocation aiming to minimise inbreeding at the genome level to be achieved more precisely, and the individual animals that have high proportions of ROH coverage to be excluded or used less frequently in mating [16]. The distribution and the occurrence of ROH have been studied in humans [10, 11, 19, 20], cattle [13–15, 18, 21–26], pigs [27–29] and sheep [17, 30–32] but are poorly studied in some species, for example, in water buffalo.

The current study aims to estimate autozygosity in the genome of AZ and KHZ buffalo breeds, and identify ROH spots that frequently occur among the individuals. The study also examines the function of the genes located in ROH islands to identify potential selection signature regions. Moreover, the study compares F_ROH_ with other genomic methods of inbreeding estimation.

## Results

### Runs of homozygosity

**Fig. 1:**
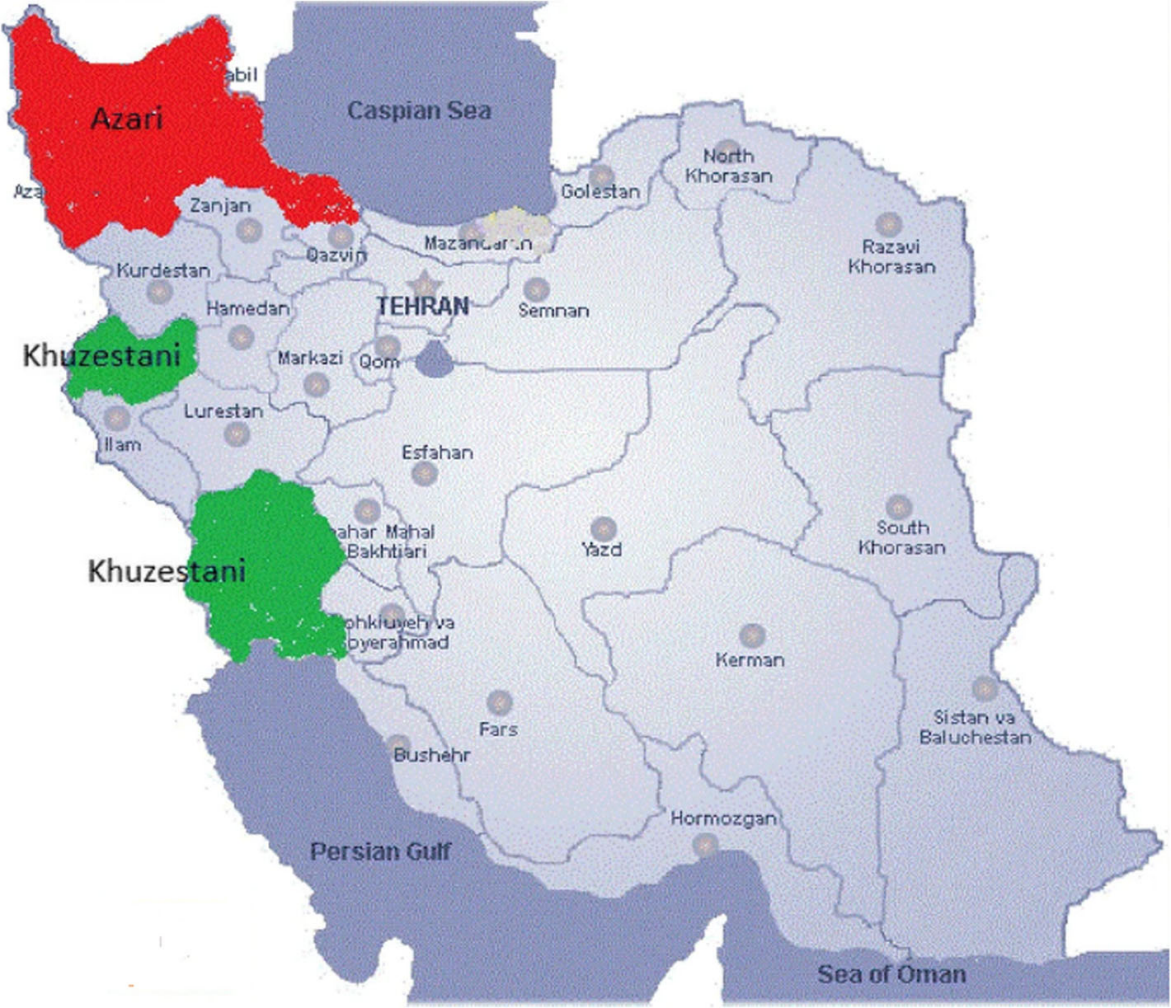
Geographic distribution of Azeri (AZ) and Khuzestani (KHZ) breeds used in this study. The samples of the AZ breed were obtained from the provinces shown in red (located in north and north-western part of Iran i.e. East and West Azerbaijan, Ardabil and Gilan). The samples for the Khuzestani (KHZ) breed were taken from the provinces shown in green (located in the west and south-western part of Iran i.e. Khuzestan and Kermanshah). Reprinted from “A genome-wide scan for signatures of selection in Azeri and Khuzestani buffalo breeds,” by Mahdi Mokhber et al., 2018; BMC Genomics., 19(1), 449. Copyright 2018 by the Creative Commons Attribution 4.0 International License (http://creativecommons.org/licenses/by/4.0/). Reprinted with permission

**Fig. 2:**
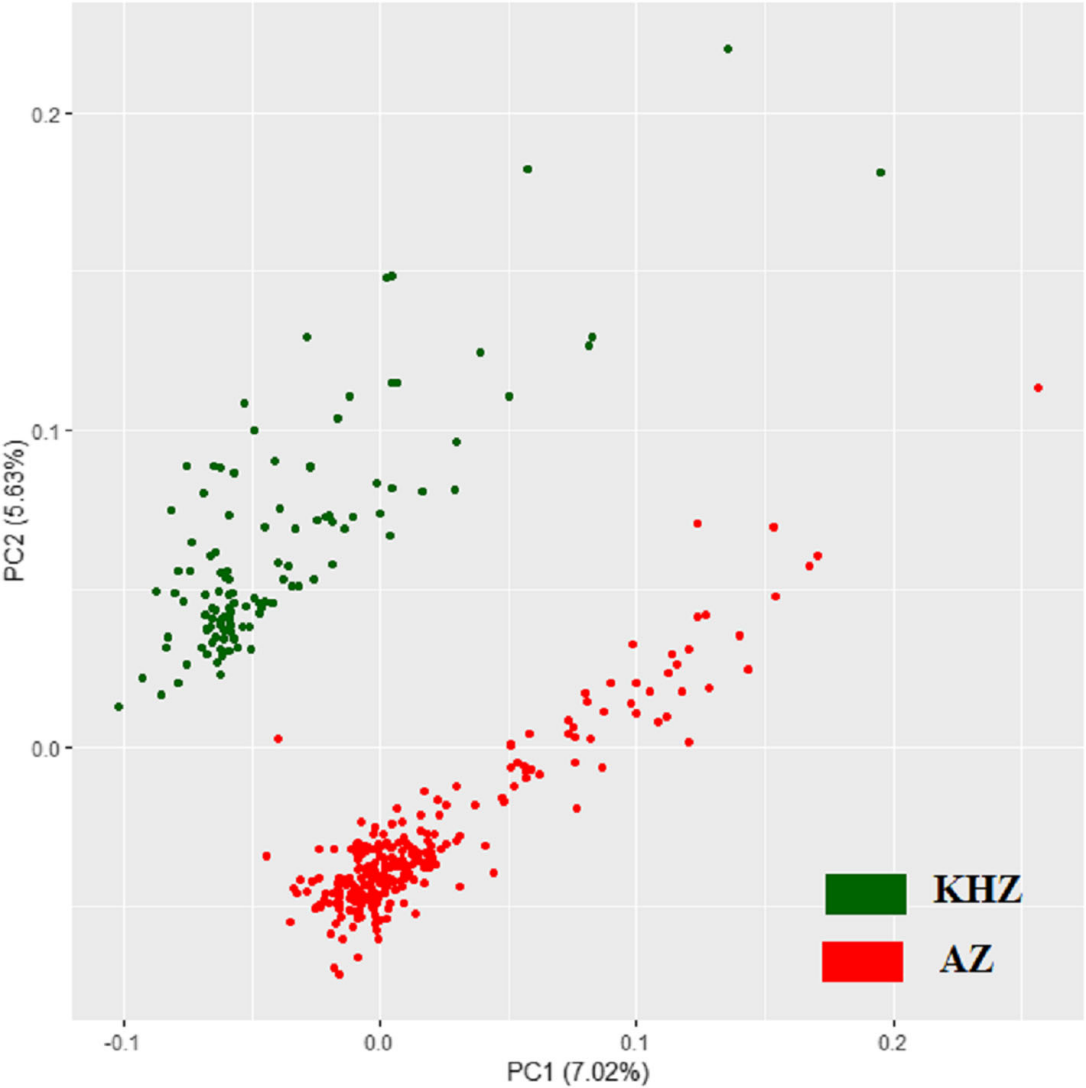
Azeri (AZ) and Khuzestani (KHZ) breeds clustered according to principal component (PC) analysis of identical by state (IBS) distance matrix. The first and second PCs explain 7.02 and 5.63% of the total variance, respectively

**Fig. 3:**
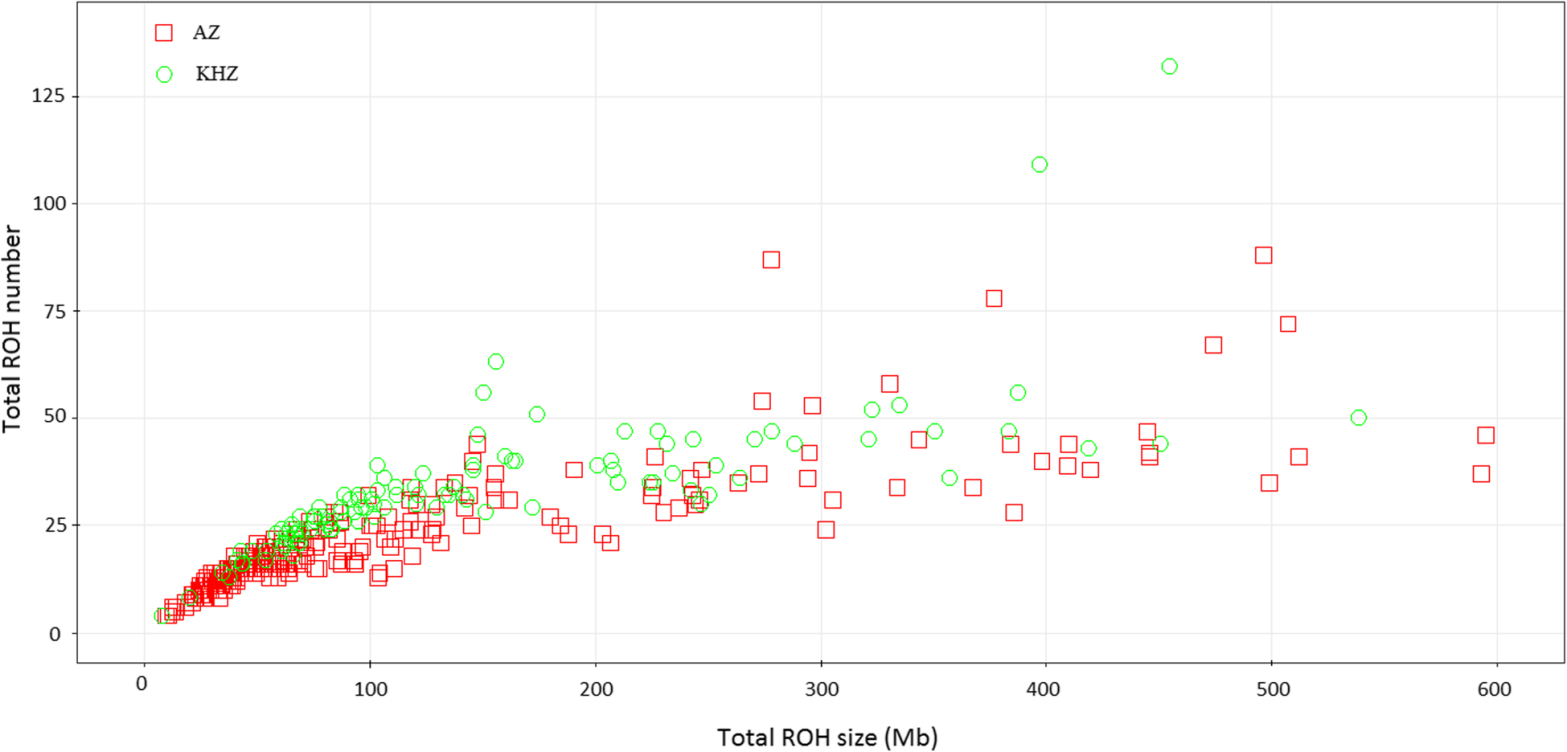
Number of runs of homozygosity (ROH) and the length of the genome covered by ROH in the samples taken from the Azeri (AZ) and Khuzestani (KHZ) breeds

The AZ and KHZ are two major buffalo breeds adapted to distinct geographical areas in Iran [2, 5] (Fig. 1). The PC analysis of the IBS matrix derived from SNP data confirmed two separate populations with no overlap, which means that the samples from the AZ and KHZ breeds were genetically different (Fig. 2). Although Mokhber et al. [5] reported that AZ and KHZ are two distinct populations, they reported a moderate level of admixture between the AZ and MZ breeds. Thus, we excluded the MZ breed from our study. In total, 9102 ROH were detected, 5352 ROH in the AZ genome and 3750 in the KHZ genome (Table 1; Additional file 1). The average number of ROH per individual was 21.23 ± 13.06 in the AZ breed (ranging from 4 to 88) and 33.2 ± 15.92 in the KHZ breed (ranging from 4 to 132). Moreover, all of the individuals in our study had at least four ROH longer than 1 Mb. The variation between samples in total number of ROH and total length of ROH are presented in Fig. 3. Individuals with an almost equal portion of the genome covered by ROH had different numbers and lengths of ROH, which could be an indication of different combinations of recent and distant inbreeding events in the samples.

**Table 1:** Summary of the detected runs of homozygosity (ROH) grouped according to their length (Mb)

ROH group	nROH^a^		Percentage		Average length (Mb)		Standard deviation (Mb)		Percentage of genome coverage	
	AZ	KHZ	AZ	KHZ	AZ	KHZ	AZ	KHZ	AZ	KHZ
ROH_1–2_	841	629	15.71	16.77	1.83	1.81	0.13	0.15	0.06	0.05
ROH_2–4_	2870	2119	53.62	56.51	2.70	2.71	0.53	0.53	0.31	0.23
ROH_4–8_	855	609	15.98	16.24	5.44	5.38	1.09	1.08	0.19	0.13
ROH_8–16_	484	241	9.04	6.42	11.23	11.22	2.25	2.21	0.22	0.11
ROH_> 16_	302	152	5.64	4.05	26.70	26.19	11.06	9.32	0.32	0.16

^a^Number of runs of homozygosity segments

### Evaluation of different methods of genomic inbreeding

Table 2 presents the averages of the estimated inbreeding coefficients using different methods (see also Additional file 2). The average F_ROH_ calculated from ROH > 1 Mb in length was 0.043 ± 0.05 in the AZ breed and 0.059 ± 0.04 in the KHZ breed (Table 2; Additional file 1). The estimated inbreeding values based on F_HOM_, F_UNI_ and F_GRM_ were higher in AZ than they were in KHZ, which was in contrast to the F_ROH_ estimates. However, the Pearson’s correlations between F_ROH_ and the estimated inbreeding with other methods were high (Table 3).

**Table 2:** Average inbreeding coefficients (± standard error) estimated using diagonal elements of genomic relationship matrix (F_GRM_), excess of homozygosity (F_HOM_), correlation between uniting gametes (F_UNI_) and runs of homozygosity (ROH) > 1 Mb (F_ROH_) in Azeri (AZ) and Khuzestani (KHZ) breeds

Breed	F_GRM_	F _HOM_	F_UNI_	F_ROH_
AZ	0.026 ± 0.05	0.026 ± 0.0.05	0.026 ± 0.05	0.043 ± 0.05
KHZ	0.019 ± 0.04	0.021 ± 0.06	0.021 ± 0.05	0.059 ± 0.04

**Table 3:** Correlation between inbreeding coefficients calculated using runs of homozygosity (ROH) > 1 Mb (F_ROH_) and estimated using diagonal elements of genomic relationship matrix (F_GRM_), excess of homozygosity (F_HOM_), and correlation between uniting gametes (F_UNI_) in Azeri (AZ) and Khuzestani (KHZ) breeds

Breed	Correlation coefficient		
	F_GRM_ -F_ROH_	F_HOM_-F_ROH_	F_UNI_-F_ROH_
AZ	0.88	0.92	0.98
KHZ	0.78	0.93	0.94

### Candidate genes inside frequently occurring runs of homozygosity regions

**Fig. 4:**
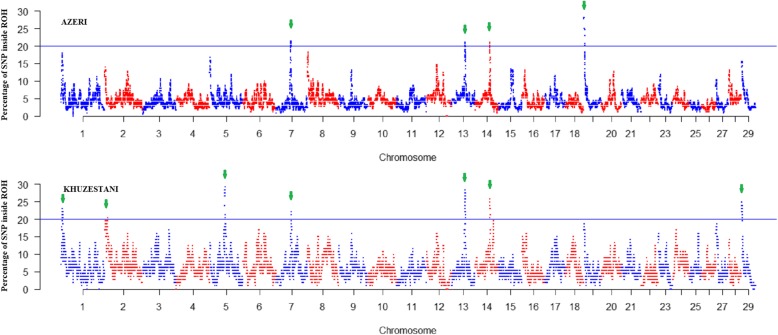
Manhattan plot of the distribution of frequently occurring runs of homozygosity (ROH) in Azeri (AZ) and Khuzestani (KHZ) Iranian water buffalo breeds. The X-axis shows the distribution of ROH over the genome, and the Y-axis shows the percentage of ROH shared among animals within each breed. The significance threshold of 20% (less than 1% of all SNPs) shown as a blue line is used for detecting ROH islands (green arrows)

A genome-wide search for SNPs that have frequently occurred within ROH hotspots revealed 11 regions on BTA1, BTA2, BTA5, BTA7, BTA13, BTA14, BTA19 and BTA29 (Fig. 4; Additional file 3). The detected ROH islands on BTA7, BTA13 and BTA14 were partially overlapped in AZ and KHZ. The strongest peaks detected in approximately 30% of the individuals were located on BTA19 (19:411,773–3,701,223 bp) in AZ and on BTA5 (5:55,217,391–57,476,442 bp) in KHZ. In the KHZ breed, the genes located in the ROH islands were significantly enriched (*P* ≤ 0.05) in 40 GO terms. These GO terms belonged to 23 biological processes (BP), 12 cellular component (CC) and 5 molecular function (MF) groups (Additional file 4).

### Co-location of ROH islands and the identified selection signatures using iHS

The majority of ROH hotspots detected in our study (Fig. 4; Additional file 3) overlapped with selection signature regions reported by Mokhber et al. [5] for the AZ and KHZ breeds using the haplotype-based method (i.e. iHS) (Additional files 5 and 6). For example, the SNP Affx-79610232 on BTA5 (55,271,590 bp) with the highest iHS was located in our detected ROH island in the KHZ breed. On BTA13, the eight SNPs with the largest iHS values were located in our detected ROH region. The SNP Affx-79540796 on BTA14 (52,933,269 bp) had the second-highest iHS value in our reported ROH hotspot. On BTA29, the SNP Affx-79545556 (3,274,219 bp) was the SNP with the sixth-highest iHS value that was located in our reported ROH islands.

## Discussion

We defined ROH as the lengths of homozygous genotypes that were > 1 Mb and contained only up to one heterozygous genotype. Given the strong linkage disequilibrium (LD) between SNPs with a distance up to 100 Kb [33], short homozygous haplotypes are expected to be prevalent in the buffalo genome. Thus, we set a minimum length of 1 Mb and a minimum number of 40 (AZ) and 38 (KHZ) SNP (as described in methods section) to avoid detecting small and prevalent haplotypes as ROH. Unlike human populations, livestock species generally have higher levels of autozygosity and longer ROHs [13, 20, 21]. However, genotyping errors can always affect the quality of ROH calling [12]. Therefore, we allowed one heterozygous SNP in ROH [25, 30, 31] to avoid losing particularly long ROH because of a single genotyping error.

As presented in Table 1, more than 53% of the detected ROH were 2–4 Mb in length. The proportion of different lengths of ROH can be used as an indicator of the number of past generations in which inbreeding has occurred, because the recombination events can rearrange the chromosomes and reduce the length of ROH. Thus, recent inbreeding results in longer ROH because of long IBD stretches. In contrast, short ROHs arise as a result of ancient inbreeding because in meiosis across generations, the long IBD segments are broken down [19]. We detected ROH with a length from 2 to 4 Mb in all of the samples (Additional file 1), which might indicate that some inbreeding events occurred about 20 generations ago [9]. However, our results should be interpreted with caution. As reported by Ferenčaković et al. [34], a medium-density chip could result in overestimation of the number of long-length ROHs (> 4 Mb), probably because some heterozygous genotypes tend to appear in these ROHs by increasing the density of markers. Nevertheless, our results were in line with a previous report of a relatively sharp decrease in the effective population size (N_e_) of AZ and KHZ breeds and the consequent increased rate of inbreeding since 20 generations ago [33].

The portion of the genome that was autozygote in the AZ and KHZ breeds was lower than the reported ROH coverage in the Marchigiana beef breed (7%) [15], Austrian dual purpose breeds (9%) [35], and Holstein cattle (10%) [36]. This could be because of lower inbreeding in Iranian water buffalo or because we ignored ROH of < 1 Mb in length in our study.

On average, F_HOM_, F_UNI_ and F_GRM_ were higher in AZ than they were in KHZ. However, the previously reported N_e_ for AZ (477) was larger than it was for KHZ (212) [33]. Therefore, we expected a lower inbreeding level in AZ. The only comparable estimated inbreeding with our expectation was F_ROH_, which showed lower inbreeding for AZ (0.043) than for KHZ (0.059).

The highest correlation was observed between F_UNI_ and F_ROH_ (AZ = 0.98 and KHZ = 0.94). Literature has reported different correlation coefficients between F_UNI_ and F_ROH_ (0.15–0.80) [14], between F_HOM_ and F_ROH_ (0.06–0.95) [14, 21, 27], and between F_GRM_ and F_ROH_ (0.17–0.81) [21, 37, 38]. The considerable variation among different studies may be because of a strong dependency of F_HOM_, F_UNI_ and F_GRM_ on allelic frequencies [39].

The F_PED_ of 0.03 previously reported in Iranian buffalo [40] was lower than the estimated F_ROH_ in the current study. Given that pedigree data were not available for our study, we could not calculate F_PED_ and compare it with F_ROH_. However, previous studies reported moderate to high (0.47–0.82) and low to moderate (0.12–0.76) correlations between F_PED_ and F_ROH_ in cattle and sheep, respectively [14, 17]. A low to moderate correlation between F_PED_ and F_ROH_ was also reported by Peripolli et al. [13] in Gyr cattle, suggesting that F_PED_ may not accurately capture small IBD segments that result from ancient inbreeding. Further, accurate and in-depth pedigree records are required to measure F_PED_. Additionally, methods based on allelic frequency have demonstrated considerable variation among different breeds [14]. Given that ROH does not depend on allele frequencies, and can capture recent and ancient inbreeding, it seems to be a suitable method for measuring inbreeding.

The total length of ROH islands were about 6 and 15 Mb in the AZ and KHZ breeds, respectively (Additional file 3). Consequently, fewer genes were identified in ROH islands in the AZ breed than in the KHZ breed; that is probably why the genes located in ROH islands of the AZ breed were not enriched in any GO terms (*P* > 0.05). In the KHZ breed, however, the genes located in the ROH islands were significantly enriched (*P* ≤ 0.05) in 40 GO terms (Additional file 4). These GO terms belonged to 23 biological processes (BP), 12 cellular component (CC) and 5 molecular function (MF) groups. In this paper, we focused principally on the GO terms that include the genes with known large effects on important traits in livestock.

Five genes were identified with positive regulation of DNA metabolic development (GO:0051054) in the BP group. Among these genes, STAT6 (signal transducer and activator of transcription 6, on BTA5) has been reported to have large effects on the growth efficiency and the quality of carcass in cattle [41]. Additionally, using co-expression network analysis, Nguyen et al. [42]. reported the critical role of STAT6, PBX2 (PBX homeobox2) and PBRM1 (Protein polybromo1) as transcription factors in regulating pubertal development in Brahman heifers.

Twelve genes in ROH islands were associated with lipid metabolic process (GO:0006629) in the BP group. Of these genes, BMP2 (bone morphogenetic protein 2, on BTA13) plays a major role in rebuilding hair follicles in goats [43]. Further, BMP2 in porcine, cattle and sheep has been reported to have an influence on regulating body size and muscle development [44–47]. Kim et al. [48] found several signatures of selection containing genes such as BMP2 associated with body size and development in goats and sheep native to Egypt. These researchers concluded that the genes influencing body size may be important in regulating adaptation to hot, arid habitats because efficiency in thermoregulation can be associated with body size. Supporting their conclusion is the fact that most breeds in tropical zones have smaller body size than breeds in temperate zones because tropical breeds can regulate their body temperature more efficiently [49]. However, other factors that differ between temperate and arid zones may also contribute to variations in the body sizes of breeds living in different climates.

CYP27B1 (cytochrome P450 family 27 subfamily B member 1) located on BTA5 was also one of the genes enriched in the lipid metabolic process (GO:0006629). This gene is important for making 1-α-hydroxylase, which is required in vitamin D bio-activation, and has been reported to be up-regulated as a result of bacterial infection, suggesting that this gene plays a role in modulating innate immune responses [50].

In ROH islands on BTA13, three genes were associated with the positive regulation of DNA replication (GO:0045740) in the BP group. Proliferating cell nuclear antigen (PCNA) has been reported to be associated with follicular development and growth in buffalo ovaries [51], and may therefore be related to fertility performance.

Single-organism cellular process (GO:0044763) with 64 genes was significantly enriched (*P* = 0.05) in ROH islands, including MARS (methionyl-tRNA synthetase, on BTA5), and ADRA1D (adrenoceptor alpha 1D, on BTA13). MARS has been reported to influence milk and protein production in Chinese [52] and Portuguese [53] Holstein cattle, and ADRA1D largely affects milk protein in Murrah dairy buffalo [54]. INHBC and INHBE (inhibin beta C and E subunits, on BTA5) have been reported as candidate genes associated with reproductive performance in tropical young bulls [55], and composite reproductive traits in Lori-Bakhtiari sheep [56]. KCTD16 (potassium channel tetramerization domain containing 16, on BTA7) was reported as a candidate gene for meat quality in Simmental beef cattle [57], for residual feed intake in Junmu White pigs [58], and for fat yield in Nordic Holstein cattle [59]. PMEL (premelanosome protein) and MYO1A (myosin IA) on BTA5 have been reported as putative candidate genes related to coat colour phenotypes in cattle [60, 61]. PMEL is required for the melanin biosynthesis process in the pigmentation of hair, mucous membranes and eyes [62]. In cattle, PMEL is reported as a candidate gene associated with the dilution of coat colour and consequently colour intensity [63, 64]. Light coat colouring can be beneficial for animals in adapting to hot climates because it can help them to reduce sunlight absorption [65]. However, most of the AZ and KHZ buffalo have a dark coat, which could be a result of some other favourable traits associated with a darker coat colour or the result of artificial selection caused by human interference. SUOX (Sulphite oxidase, on BTA5), within this BP category, was reported to be associated with bone development in cattle [66].

The average LD (r^2^) between adjacent SNPs in ROH islands was higher than the r^2^ of adjacent SNPs located on the same chromosome (Additional file 3). Thus, the recombination rates in the ROH islands were lower than those in the rest of the genome. These results are in line with some previous studies [13, 17]. However, a moderate recombination rate has been reported between the SNPs in ROH islands in Valle del Belice sheep [67]. Additionally, ROH hotspots can result from a wide range of underlying causes such as inbreeding and selection [12]. Peripolli et al. [13] argued that the high LD observed in most ROH hotspots is not necessarily caused by selection or conserved IBD haplotypes, but can be an indication of a lower recombination rate in those regions. Nevertheless, most of the ROH hotspots in our study overlapped with selection signature regions found with iHS, which supports the theory that ROH can be used to find genomic regions that have been under natural and/or artificial selection.

Buffalo species have a relatively lower heat tolerance capability than some other livestock species because of their inadequately dispersed sweat glands and their dark coat colour [68]. However, Iranian buffalo breeds have historically been raised in a hot climate [69]. Therefore, selection for higher heat tolerance may have occurred in Iranian buffalo for better adaptation to heat stress [5]. It has been reported that combined networks of multiple genes are often involved in the regulation of complex traits such as adaptation to hot climates [48, 70, 71]. Thus, selection for complex traits would leave only minor footprints because of the selection for numerous regions with lower intensity across the genome [70]. Therefore, we expected to find several genes directly or indirectly influencing different traits that were under artificial selection or important for adaptation and survival in hot areas. We found genes influencing energy and digestive metabolism (KCTD16), autoimmune response (CYP27B1), thermoregulation (BMP2), embryonic development and reproduction (STAT6, PCNA, INHBC and INHBE). These genes seem to be important for species such as water buffalo that have adapted to a hot climate [48].

## Conclusion

The inbreeding coefficients based on F_HOM_, F_UNI_ and F_GRM_ were higher in the AZ breed than they were in the KHZ breed, which contradicted our expectations according to higher N_e_ in AZ breed. Given that F_ROH_ was the only measurement of inbreeding in our study that showed KHZ water buffalo were more inbred, this measurement seems to be a suitable measure of genomic inbreeding. This is most likely because it is less affected by allele frequencies. Further, knowing the distribution of ROH across the genome, inbreeding can be avoided more efficiently through mating allocation. Additionally, frequently occurring ROH can be used as suggestive evidence of historical selection. In our study, we found some overlap between ROH islands and genomic regions showing signatures of selection in previous studies of AZ and KHZ breeds. Therefore, the genes located in ROH islands could be under the influence of artificial and/or natural selection. We found that the genes located in ROH islands were associated with biological pathways such as adaptation to a hot climate, immune response, milk production, growth efficiency, reproduction performance and bone development.

## Methods

### Sample collection, ethical statement, and data quality control

Hair roots and blood samples were obtained from 112 herds of AZ and 47 herds of KHZ breeds. Samples of the AZ breed were gathered from East and West Azerbaijan, Gilan and Ardabil (37.02° – 38.78° N, 44.81° 49.52°E), which are north-western provinces of Iran. Samples of the KHZ breed were obtained from Kermanshah (34.54°N, 45.60°E) and Khuzestan (30.68–32.55° N, 48.02°–48.97° E), which are the south and south-western provinces of Iran, respectively (Fig. 1). All practices relating to data collection were reviewed and confirmed by the research ethics committee of the College of Agriculture and Natural Resources of the University of Tehran, Iran and by the ABCI. Three hundred and sixty-nine buffalo (254 AZ and 115 KHZ) were genotyped using 90 K SNPChip (Axiom® Buffalo 90 K Genotyping Array), which consisted of 89,988 almost evenly distributed SNPs throughout the genome. The same dataset was previously used by Mokhber et al. [5], and it partially overlapped with the dataset used by Colli et al. [4] and by Fallahi et al. [72].

The SNPs in the 90 K SNPChip were selected using buffalo DNA sequence, but similar to methods used in previous studies [1, 5, 33, 72–75], were reported according to the location on the cattle reference genome assembly (UMD3.1 [76]) Although chromosome-level assembly of the water buffalo genome (UOA_WB_1) has been published recently [77], we used the UMD3.1 assembly in our study because it is more reliable and has better gene annotation information. Genotypes were obtained through AffyPipe [78], and all the monomorphic and polymorphic SNPs with high resolution (*n* = 64,750) were stored. According to the filtration criteria, samples with more than 5% missing genotype and SNPs with 5% missing rate were eliminated from further analyses. We also filtered out SNPs with unidentified position in the UMD3.1 assembly, positioned on the sex chromosomes, with minor allele frequency of < 2%, and with *p*-value for the Hardy–Weinberg equilibrium chi-square test < 10^− 6^. In total, 62,122 SNPs and 369 samples with an average call rate of 99.6% passed the quality-control filters.

### Genetic distance between breeds

Genetic distance, which is based on the IBS matrix, was estimated through the --ibs-matrix command in PLINK v1.9 [79]. Principal component (PC) analysis of genetic distances was performed to visualise the genetic diversity of the samples, and was depicted using R (http://www.R-project.org/). According to the first and second PCs, we removed four samples: two from each breed that were placed outside their expected breed cluster.

### Runs of homozygosity analyses

ROH can be detected in the genome through two main approaches: 1) genotype-counting algorithms in which the genome is scanned to identify long stretches of consecutive homozygous genotypes like the one implemented in PLINK v1.9 [79], and 2) model-based methods that utilize Hidden Markov Models (HMM) like the one implemented in RzooRoH [80]. This package can enable a better assessment of the contribution of various generations to the current level of inbreeding, estimating inbreeding at both genome-wide and local scales, and classifying homozygous-by-descent (HBD) segments into age-based classes [81]. However, we used PLINK in our study because of the simplicity of running the sliding-window approach to detect ROH with sufficiently high assurance [82].

A genome scan for ROH was conducted for the AZ (*n* = 252) and KHZ (*n* = 113) breeds, separately. For each individual, ROH segments with the following attributes were identified: 1) each ROH stretch was at least 1 Mb in length; 2) there was at the most only one heterozygous and one missing SNP in each ROH; 3) there was a minimum number of SNPs that could form ROH in each breed, calculated according to Eq. 1 to control the false positive rate of the identified ROH.

1 $$ l=\frac{{\mathit{\log}}_e\frac{\alpha }{n_a{n}_s}}{{\mathit{\log}}_e\left(1- het\right)}, $$

where *l* is the minimum number of SNPs in ROH, α is the false positive rate of the identified ROH (set at 0.5); n_a_ and n_s_ are the number of individuals and the number of SNPs per individual, respectively; and *het* is the average heterozygosity across individuals. *l* was calculated to be 40 and 38 in AZ and KHZ breeds, respectively; 4) each ROH contained at least one SNP over 100 Kb; and 5) the maximum gap between two neighbouring SNPs in ROH had to be less than 1 Mb.

The ROH that had these five attributes were divided into the following five groups: 1–2, 2–4, 4–8, 8–16 and > 16 Mb, as suggested in the literature [13, 15, 25]. Then for each breed, the frequency and the average length (Mb) of ROH within each category, the percentage of each ROH category, and the percentage of genome coverage by each ROH category were calculated, using R (http://www.R-project.org/).

### Inbreeding coefficient estimations

The coefficient of inbreeding was estimated using ROH (F_ROH_), excess of homozygosity (F_HOM_), correlation between uniting gametes (F_UNI_) and diagonal elements of the genomic relationship matrix (F_GRM_).

F_ROH_ was calculated for each individual using Eq. 2 [20]:

2 $$ {\mathrm{F}}_{ROH_i}=\frac{\sum_{j=1}^n{L}_{ROH_j}}{L_{aut}}, $$

where ${F}_{{\mathrm{ROH}}_i}$ is the inbreeding coefficient of animal *i*; *n* is the total number of ROH; and ${\mathrm{L}}_{{\mathrm{ROH}}_{\mathrm{j}}}$ is the length of the *j*^th^ ROH in animal *i*; *L*_aut_ is the total autosome length covered by the SNP markers (2.5 Gb in our study).

We also calculated the following three different genomic inbreeding estimations: F_GRM_ (Eq. 3), F_HOM_ (Eq. 4) and F_UNI_ (Eq. 5) using --ibc command in GCTA software [39].

3 $$ {\mathrm{F}}_{GRM}=\frac{1}{n}{\sum}_{i=1}^n\frac{{\left({x}_i-2{p}_i\right)}^2}{h_i}-1, $$

4 $$ {\mathrm{F}}_{Hom}=1-\frac{1}{n}{\sum}_{i=1}^n\frac{x_i\left(2-{x}_i\right)}{h_i}, $$

5 $$ {\mathrm{F}}_{UNI}=\frac{1}{n}{\sum}_{i=1}^n\frac{x_i^2-\left(1+2{p}_i\right){x}_{\mathrm{i}}+2{p}_i^2}{h_i}, $$

where *x*_*i*_ and *p*_i_ are the number of copies and the frequency of the reference allele for SNP *i*, respectively; *h*_i_ is 2*p*_i_(1–2*p*_i_); and n is the total number of SNPs. The Pearson’s correlation coefficient between F_ROH_ and the other genomic inbreeding estimates was also calculated.

### Frequently appearing runs of homozygosity and gene enrichment analyses

To detect the genomic regions frequently covered with ROH in the AZ and KHZ populations, the number of times each SNP occurred in ROH was calculated separately in each breed. The ROH repeated in more than 20% of the individuals in each breed (approximately less than 1% of the SNPs) were nominated ROH islands, as suggested in previous studies [25, 67]. Further, the frequency of ROHs were plotted against their physical position along UMD3.1.

To identify genes in ROH islands, we used UMD3.1 map viewer from the NCBI website (https://www.ncbi.nlm.nih.gov/mapview/). Additionally, to find significantly enriched Gene Ontology (GO) terms (*P* ≤ 0.05) of the genes located in ROH peaks, we used DAVID v6.8 tool [83, 84]. Finally, we performed an extensive literature review to explore the biological function of the annotated genes in ROH islands.

To discover whether ROH islands were associated with regions of the genome with a low recombination rate, the average LD of all the adjacent SNPs across each chromosome was compared with the average LD between adjacent SNPs inside the ROH islands located on the same chromosome. Additionally, to discover whether the ROH hotspots were associated with genomic regions that showed signatures of selection through other methods, we compared ROH islands with integrated haplotype homozygosity scores (iHS) that had already been published for AZ and KHZ breeds [5]. An iHS is a measure of haplotype homozygosity based on the difference between observed LD structure around a selected allele relative to the expected LD pattern according to the whole genome [85]. Therefore, it can be used to detect regions under historical selection [85].

## Supplementary information

**Additional file 1.** List of the detected runs of homozygosity (ROH) in Azeri (AZ) and Khuzestani (KHZ) breeds.

**Additional file 2.** The estimated inbreeding coefficient using runs of homozygosity (ROH) > 1 Mb (F_ROH_), diagonal elements of genomic relationship matrix (F_GRM_), excess of homozygosity (F_HOM_) and correlation between uniting gametes (F_UNI_) in Azeri (AZ) and Khuzestani (KHZ) breeds.

**Additional file 3.** Frequently occurring runs of homozygosity (ROH) regions (i.e. ROH islands) in Azeri (AZ) and Khuzestani (KHZ) breeds. The last column represents the average r^2^ of ROH islands divided by the average r^2^ of each chromosome.

**Additional file 4. **The genes located in detected runs of homozygosity (ROH) islands in the Khuzestani (KHZ) breed that were significantly enriched (*P* ≤ 0.05) in biological processes (BP), cellular component (CC) and molecular function (MF) Gene Ontology (GO) terms.

**Additional file 5.** List of integrated haplotype homozygosity scores (iHS) for all SNPs in Azeri (AZ) and Khuzestani (KHZ) breeds.

**Additional file 6.** Manhattan plot of integrated haplotype homozygosity score (iHS) across the genome.

## Data Availability

All data generated or analyzed during this study are included in this published article and its additional files.

## Abbreviations

ABCI: Animal Breeding Centre of Iran
AZ: Azeri breed
F_GRM_: Inbreeding coefficient calculated using diagonal elements of the genomic relationship matrix
F_HOM_: Inbreeding coefficient calculated using excess of homozygosity
F_ROH_: Inbreeding coefficient calculated using ROH
F_UNI_: Inbreeding coefficient calculated using correlation between uniting gametes
GO: Gene Ontology
IBD: Identical by descent
IBS: Identical by state
iHS: Integrated haplotype homozygosity score
KHZ: Khuzestani breed
LD: Linkage disequilibrium
MZ: Mazandarani breed
N_e_: Effective population size
PC: Principal component
ROH: Runs of homozygosity
SNP: Single nucleotide polymorphism
