# Correction to: Genomic measures of inbreeding coefficients and genome-wide scan for runs of homozygosity islands in Iranian river buffalo, *Bubalus bubalis*

**DOI:** 10.1186/s12863-020-00841-7

**Published:** 2020-04-08

**Authors:** Seyed Mohammad Ghoreishifar, Hossein Moradi-Shahrbabak, Mohammad Hossein Fallahi, Ali Jalil Sarghale, Mohammad Moradi-Shahrbabak, Rostam Abdollahi-Arpanahi, Majid Khansefid

**Affiliations:** 1 Department of Animal Science, University College of Agriculture and Natural Resources, University of Tehran, Karaj, 31587-11167 Iran; 2 Departments of Animal and Poultry Science, College of Aburaihan, University of Tehran, Pakdasht, 33916-53755 Iran; 3 AgriBio Centre for AgriBioscience, Agriculture Victoria, Bundoora, VIC 3083 Australia

**Correction to: BMC Genet**

**https://doi.org/10.1186/s12863-020-0824-y**

Following publication of the original article [1], the authors flagged that the article had published with the author ‘Ali Jalil Sarghale’ erroneously omitted from the author list.

The author list has now been corrected in the original article.

Please also find the corrected author list in this correction article.

The publisher apologizes for this processing error.
