# Supplementary figures and images for: Genomic measures of inbreeding coefficients and genome-wide scan for runs of homozygosity islands in Iranian river buffalo, Bubalus bubalis
Source: BMC Genet. 2020 Feb 10;21:16. doi: 10.1186/s12863-020-0824-y (PMC7011551; doi:10.1186/s12863-020-0824-y)

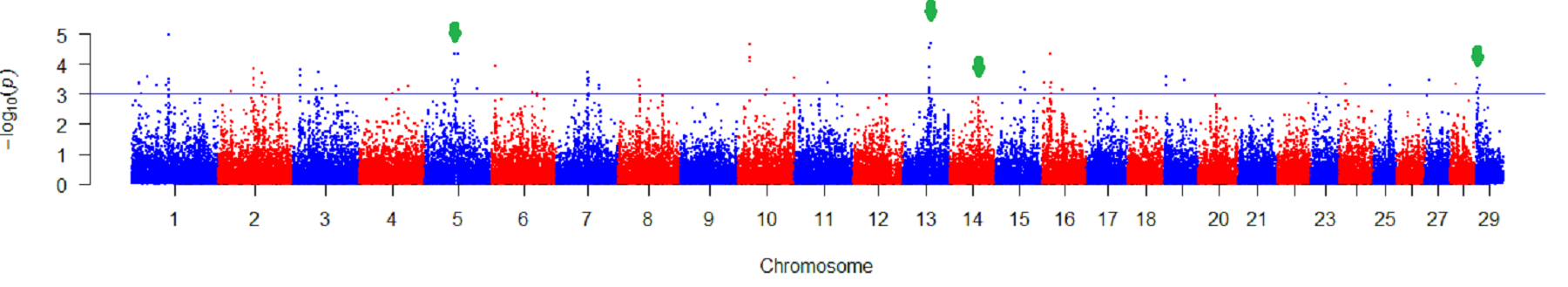

Supplement: Supplementary file 6 — Additional file 6. Manhattan plot of integrated haplotype homozygosity score (iHS) across the genome. [file 12863_2020_824_MOESM6_ESM.tif]
